# Insights into the Regulatory Effect of Danggui Buxue Tang in Postpartum Dairy Cows Through an Integrated Analysis of Multi-Omics and Network Analysis

**DOI:** 10.3390/life15030408

**Published:** 2025-03-05

**Authors:** Kang Yong, Zhengzhong Luo, Zheng Zhou, Yixin Huang, Chuanshi Zhang, Suizhong Cao

**Affiliations:** 1 College of Animal Science and Technology, Chongqing Three Gorges Vocational College, Chongqing 404155, China; 2 College of Veterinary Medicine, Sichuan Agricultural University, Chengdu 611130, China; 3 Key Laboratory of Animal Disease and Human Health of Sichuan Province, Chengdu 611130, China

**Keywords:** postpartum dairy cows, Danggui Buxue Tang, prototype metabolites, metabolic adaptation, gut microbiota

## Abstract

Postpartum dairy cows often face significant challenges due to metabolic disorders. Danggui Buxue Tang (DBT), a botanical drug composed of *Astragali radix* and *Angelica sinensis radix* in a 5:1 ratio, has been recognized for its potential to alleviate metabolic disorders. Its regulatory mechanisms on livestock metabolic health have remained unexplored. This study integrated the analyses of serum pharmacochemistry, network pharmacology, serum metabolomics, and fecal microbiota to investigate the regulatory effects of DBT on metabolic adaptation in postpartum dairy cows. Following the oral administration of DBT, levels of blood non-esterified fatty acids and beta-hydroxybutyrate were decreased in multiparous dairy cows one week after calving. Five absorbed prototype metabolites of DBT were identified, specifically formononetin and nicotinic acid, both of which play roles in the regulation of lipid metabolic homeostasis. Furthermore, DBT modified the composition of the gut microbial community and glycerophospholipid levels. Decreases in serum phosphatidylethanolamine and phosphatidylcholine levels were closely correlated with the relative abundance of *Bacillus* and the concentration of circulating beta-hydroxybutyrate. These findings suggest that DBT contributes positively to metabolic health in postpartum dairy cows by regulating the gut microbiota and glycerophospholipid metabolism, providing new insights into strategies for promoting metabolic adaptation in dairy cows.

## 1. Introduction

Transitioning dairy cows suffer from considerable challenges related to metabolic abnormalities, particularly the metabolic disorders that typically manifest in the postpartum period [1,2]. Early lactation in dairy cows with metabolic disorders is closely linked to the development of various diseases, including ketosis, left-displaced abomasum, and hypocalcemia [3]. Significant elevations in circulating non-esterified fatty acids (NEFA) and beta-hydroxybutyrate (BHB) levels are characteristic indicators of metabolic disorders in postpartum dairy cows [2,4]. Typically, dairy cows adapt to the increased energy requirements after calving through enhanced lipid mobilization, which is accompanied by a rapid increase in circulating NEFA level [5]. Non-esterified fatty acids are transported to the liver and metabolized to BHB via the beta-oxidative pathway. Moreover, elevated NEFA levels can lead to metabolic inflammation and impaired insulin signaling [6,7]. Furthermore, NEFA and BHB serve as markers of postpartum disease risk in dairy cows [8]. Therefore, it is crucial to alleviate lipid mobilization in dairy cows after calving to improve their metabolic health.

Danggui Buxue Tang (DBT) is a traditional Chinese medicinal formula composed of the dried roots of *Astragali* and *Angelica sinensis* in a 5:1 ratio. The “Treatise on Febrile Diseases” by Dongyuan Li was the first to describe DBT as a medicine that supported “Qi” (energy) and enriched blood. Recent studies have confirmed the pharmacological effects of DBT in the regulation of metabolic disorders [9,10]. In addition, DBT can modulate BHB metabolism and suppress oxidative stress [11]. Some studies on the potential mechanisms of action indicate that DBT positively influences health by remodeling the gut microbiota [10,12]. Gut microbiota play a significant role in regulating the development of metabolic diseases [13]. In regard to dairy cows, recent studies have shown that the composition and function of the gut microbiota correlate with excessive lipolysis and oxidative stress during the transition period [14,15]. Previous studies have also demonstrated that alterations in the gut microbiota can contribute to excessive lipolysis and insulin insensitivity in dairy cows during the development of ketosis [16]. Although benefits function as DBT on gut microbial community balance and metabolic health, the regulatory influence on dairy cows remains unknown.

With extensive use of liquid chromatography–tandem mass spectrometry (LC-MS), the chemical compounds of DBT, including astragaloside, formononetin, ferulic acid, senkyunolide, and isoastragaloside, have been determined [17]. However, the unique digestive physiology of ruminants may alter the bioavailability and efficacy of herbal compounds, thereby underscoring the critical need for species-specific pharmacological investigations. Unlike prior applications focused on non-metabolic contexts, this study hypothesizes that DBT’s synergistic components (e.g., formononetin and nicotinic acid) may mitigate lipid mobilization while modulating gut microbial community and glycerophospholipid metabolism, thereby reducing metabolic stress of postpartum cows. Furthermore, adapting this well-established human tonic to livestock systems aligns with the growing demand for natural alternatives to synthetic additives, offering a sustainable strategy to improve dairy cattle health and productivity. In this study, serum pharmacochemical and network pharmacological analyses were conducted to investigate the absorbed prototype metabolites of DBT following intervention in postpartum dairy cows. Subsequently, serum metabolomics and fecal microbiota analyses were performed using LC-MS and 16S rRNA amplicon sequencing, respectively. Finally, the associations between metabolic phenotypes, key metabolites, and gut microbiota were integrated to explore the regulatory effects of DBT on metabolic health in postpartum dairy cows.

## 2. Materials and Methods

### 2.1. DBT Preparation and Animals Management

*Astragali radix* (Huangqi in Chinese, HQ) and *Angelica sinensis radix* (Danggui in Chinese, DG) were purchased from Minxian Ronghe Pharmaceutical Co., Ltd. (Gansu, China). HQ and DG were air-dried and ground into a fine powder (particle size < 15 µm). The DBT powder was prepared based on a ratio of HQ to DG of 5:1. Formononetin, ononin, and ferulic acid were identified as quality markers for DBT, with their relative peak area percentages measuring 0.235%, 0.131%, and 0.045%, respectively.

Twelve multiparous transition dairy cows with similar body condition scores, age, and parity were selected from a commercial dairy farm in Ningxia Hui Autonomous Region, China. They were then randomly divided into either a control (n = 6) or DBT (n = 6) group. Our previous dose-optimization study established 240 g per day as the optimal DBT dosage [18]. On calving day, the DBT group cows were orally administered 240 g DBT powder mixed with 3 L of water and were then given the same treatment for the next six days. In the control group, the cows were orally administered 3 L water on calving day and given the same treatment for the next six days. All treatments were administered at 08:00.

### 2.2. Sample Collection and Serum Markers Analysis

Before morning feeding, blood samples were collected from dairy cows via the caudal vein on the day of calving (day 0) and one week after calving (day 7). The blood BHB concentration was determined using a ketone detector. Meanwhile, serum samples were obtained through centrifugation at 2000× *g* and 4 °C for 10 min. Fecal samples from all dairy cows were collected via rectal examination on days 0 and 7. Serum levels of NEFA, glucose, triglycerides, total cholesterol, total protein, and albumin were determined using commercially available kits (Nanjing Jiancheng Bioengineering Institute, Nanjing, China) following the manufacturer’s instructions.

### 2.3. Sample Pretreatment

The 100 μL of serum sample on day seven was mixed with 400 μL of methyl alcohol and subjected to ultrasound for 20 min in an ice bath. The supernatant was collected after centrifugation at 16,000× *g* for 20 min at 4 °C, and then redissolved in 50% methyl alcohol. Subsequently, 80 μL of the supernatant was collected after centrifugation at 20,000× *g* for 15 min at 4 °C. Similarly, 100 mg of DBT powder was mixed with 5 mL of 75% ethyl alcohol and ultrasonicated for 1 h at 25 °C. The resulting supernatant was collected after centrifugation at 3000× *g* for 10 min at 4 °C, and then dried using nitrogen. Ultrapure water was added to the DBT to bring the volume to 1 mL, and the mixture was then filtered using a 0.22 μm filtration membrane.

### 2.4. Serum Pharmacochemistry and Metabolomics Profiling Analyses

After pre-treatment, the serum and DBT samples underwent separation using ultra-high performance liquid chromatography (Nexera LC-40, Shimadzu, China) with an HSS T3 column (2.1 mm × 100 mm, 1.8 µm, Waters, Milford, MA, USA). The injection volume was 10 μL, and the column temperature was maintained at 40 °C. The flow velocity was set to 0.3 mL/min. Mobile phase A consisted of a 0.1% formic acid solution, whereas phase B consisted of a 0.1% formic acid–acetonitrile solution. The chromatographic gradient elution procedure was as follows: 0–5 min, 0% B; 5–20 min, a linear change from 0% to 100% B; 20–25 min, 100% B; 25–25.1 min, a linear change from 100% to 0% B; 25.1–30 min, 0% B. The samples were then analyzed using a quadrupole–orbitrap mass spectrometer (Q Exactive™ Plus, Thermo Scientific, Waltham, MA, USA) with an electrospray ionization source in positive and negative ion modes. The ionization conditions were set as follows: the capillary temperature was 320 °C, the sheath gas was set to 30, the auxiliary gas at 5, and the spray voltage was 3.8 kV in positive mode or 3.2 kV in negative mode. The precursor ion mass scan range was set to 90–1350 *m*/*z*, with a mass spectrometry resolution of 70,000 (FMHW@*m*/*z* 200). A full MS/MS scan was performed using a higher-energy collisional dissociation-activated method based on a resolution of 17,500 (FMHW@*m*/*z* 200). Graphic generation of base-peak intensity chromatograms and interpretation of mass spectra were performed using Xcalibur software (version 4.3).

The retention time and peak area of the raw MS data in DBT, control serum, and DBT serum were extracted using MS-DIAL software (version 5.2) [19]. The characteristic metabolites of the Chinese medicines were annotated using the self-constructed BP-TCM database from Shanghai BIOPROFILE Technology Co., Ltd. (Shanghai, China). Mass tolerance (MS/MS < 0.01 Da) and a matching score > 50% were the standards for metabolite identification. The constituents absorbed from the botanical drugs into the blood were identified based on previous reports [20,21,22]. Furthermore, the serum metabolic profiles of the control and DBT groups were analyzed by matching them with the self-constructed BPDB database. Metabolomics data were normalized using Log10 transformation and Pareto scaling to approximate a normal distribution and minimize variance heterogeneity. Differential metabolite analysis between the control and DBT groups was conducted using fold-change analysis and Student’s *t*-tests. Multivariate statistical analysis was performed using the *Ropls* package (version 1.38.0) in R software (version 4.4), including principal component analysis (PCA) and orthogonal partial least squares discriminant analysis (OPLS-DA). To validate the robustness of the OPLS-DA model, a permutation test (n = 1000 iterations) was conducted, with model quality assessed using R2Y and Q2 parameters.

### 2.5. Network Pharmacology and Molecule Docking

The target genes of the metabolites absorbed into the blood by DBT were analyzed using the Swiss Target Prediction platform. The terms ‘ketosis’, ‘hyperketonemia’, and ‘lipid mobilization’ were used as keywords and searched in the databases including GeneCards (https://www.genecards.org/, accessed on 15 October 2024), Online Mendelian Inheritance in Man (https://omim.org/, accessed on 15 October 2024), and DisGenet (https://www.disgenet.org/, accessed on 15 October 2024). Common target genes associated with metabolic disorders were identified and deduplicated across multiple databases. Subsequently, these targets were cross-referenced with the ‘*Bos taurus*’ genome using BLAST alignment to ensure species relevance. Venn diagram analysis was employed to identify overlapping targets between DBT’s components and cow metabolic disorders, which represent the most promising candidate targets for further investigation. The KEGG pathways of the shared target genes were annotated using the Database for Annotation, Visualization, and Integrated Discovery. Analysis of protein–protein interaction networks of shared targets was performed using the STRING database (version 12.0). Network topology analysis was performed to identify key valuable targets using the Cytoscape platform (version 3.10.2). The centrality parameters of the top 10 targets, including degree centrality, betweenness centrality, and closeness centrality, were systematically evaluated using both CytoNCA (version 2.1.6) and CytoHubba (version 0.1) plugins. Shared targets consistently ranked within the top 10 by both CytoNCA and CytoHubba were selected for subsequent molecular docking.

The 3D structures of the target compounds in DBT were obtained from PubMed (https://pubchem.ncbi.nlm.nih.gov/, accessed on 20 October 2024). The minimized conformational energy of the chemical compound was calculated using Chem3D software (version 23.0). The 3D structure of the target protein in *Bos taurus* was obtained from the AlphaFold Database (https://alphafold.com, accessed on 20 October 2024). Subsequently, molecular hydrotreatment was conducted and molecular docking between the chemical compound in the botanical drug and the protein was performed using the AutoDock tool (version 4.2.6). The docking mode diagram was generated using PyMOL software (version 3.0).

### 2.6. Fecal 16S rRNA Amplicon Sequencing and Data Pretreatment

The total genomic DNA of fecal samples on day 7 was extracted using a commercial kit. Genome quality was assessed by electrophoresis on a 1% agarose gel. The V3–V4 region of the 16S rRNA gene was amplified using the PCR system (ABI GeneAmp^®^ 9700, Thermo Fisher Scientific, Waltham, MA USA) with primers 341F (5′-CCTAYGGGRBGCASCAG-3′) and 806R (5′-GGACTACNNGGGTATCTAAT-3′). Following purification, the PCR products were quantified using the QuantiFluor™-ST fluorescence quantification system (Promega, Beijing, China). The amplicon pools were sequenced on a NovaSeq 6000 platform (Illumina, San Diego, CA, USA). Raw data processing and feature annotation of amplicon sequence variants were performed as previously described [16].

### 2.7. Statistical Analysis

The alpha and beta diversities of the fecal microbial communities were analyzed using the *vegan* package. Comparison between the control and DBT groups in serum variables was performed using Student’s *t*-test or the Mann–Whitney U test, as appropriate. Differences between the two groups in microbial abundance were quantified using Cohen’s d, where |d| > 0.2, > 0.5, and > 0.8 represent small, medium, and large effect sizes, respectively. Confidence intervals (95% CI) for relative abundance changes were calculated using the bootstrap method. Correlation analysis between fecal microbes and serum metabolites was conducted using Spearman’s coefficient. The false discovery rate was controlled using the Benjamini–Hochberg method for multiple testing correction, with statistical significance set at adjusted *p* < 0.05. Variables important in projection obtained from OPLS-DA (VIP > 1) and *p* < 0.05 were used as criteria for screening differential metabolites. The origins of differential metabolites, including host, microbiota, and co-metabolism, were analyzed using the MetOrigin 2.0 platform (https://metorigin.met-bioinformatics.cn/home/, accessed on 25 October 2024). Additionally, topology analysis of the KEGG pathways was performed using MetaboAnalyst 6.0 (https://www.metaboanalyst.ca/, accessed on 25 October 2024). The interrelationships among fecal microbes, serum metabolites, and metabolic phenotypes were visualized using the Cytoscape software (version 3.10) based on Spearman’s correlation. Data are presented as mean ± SEM, and graphs were created using GraphPad (version 10) and R (version 4.3) tools.

## 3. Results

### 3.1. Effect of DBT on Metabolic Biomarkers in Dairy Cow After Calving

On days 0 and 7, serum levels of triglycerides, total cholesterol, glucose, albumin, and total protein were not significantly different between the control and DBT groups. Among them, the glucose level had a significant effect (*P*_Time_ < 0.01) on the change in lactation days (Table 1). Compared to the control group on day 7, the levels of blood BHB (*p* < 0.01) and NEFA (*p* = 0.065) were decreased in the DBT group. Notably, the result in blood BHB level showed a significant interaction effect (*P*_Time × group_ = 0.004) between days in milk and DBT intervention. NEFA levels had a significant effect (*P*_Time_ < 0.01) on the days in milk.

### 3.2. Identification of Absorbed Prototype Metabolites in DBT

**Figure 1:**
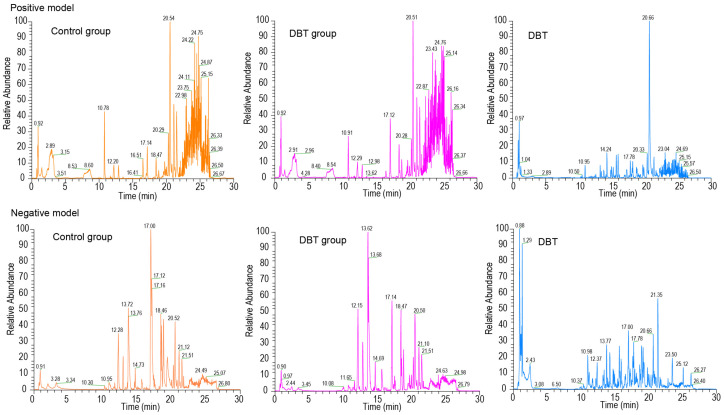
Base-peak intensity chromatograms of control group, DBT group, and DBT botanical drug samples obtained by UHPLC–MS/MS analysis in positive-ion and negative-ion modes.

The base peak intensity chromatograms showed a similar distribution of ionic peaks between the control and DBT groups in the positive mode while showing differences from the DBT botanical drug samples (Figure 1). Notably, the ionic strength between the control and DBT groups had a marked difference in the negative mode. Through LC-MS analysis, 753 metabolites were identified in DBT. Five prototype metabolites absorbed into the blood were identified in dairy cows following the oral administration of DBT: amygdalin, formononetin, kaempferol 3-glucuronide, nicotinic acid, and soyasaponin I (Table 2). It is essential to highlight that the formononetin, kaempferol 3-glucuronide, nicotinic acid, and soyasaponin I were derived from HQ.

### 3.3. Network Analysis of Prototype Metabolites in DBT

**Figure 2:**
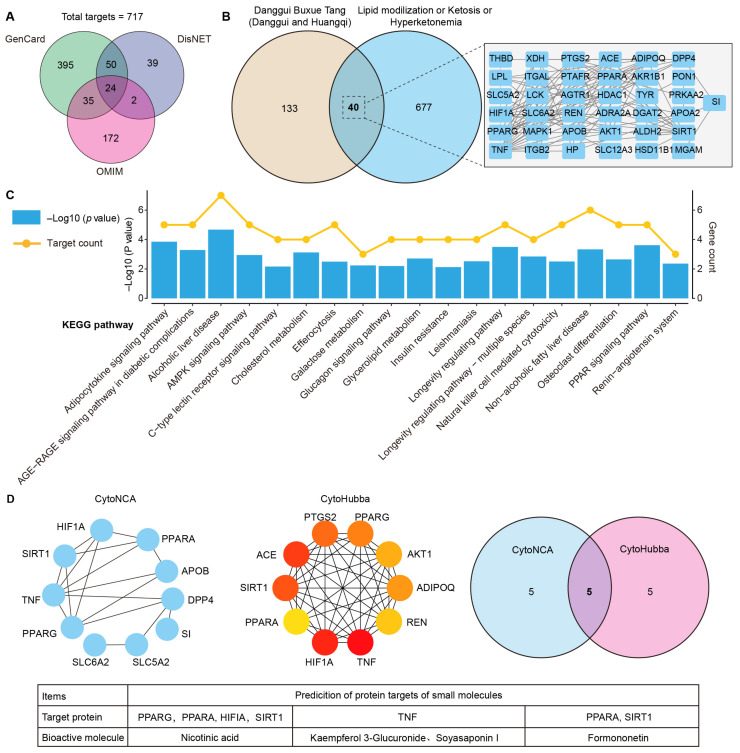
Network pharmacology analysis of absorbed prototype metabolites in DBT. (**A**) Disease targets. (**B**) Venn diagram indicates the shared targets between the botanical drug and disease targets. (**C**) KEGG pathway analysis of key targets. (**D**) Screening of interaction relationship between the key targets and prototype metabolites based on cytoNCA and cytoHubba analyses.

**Figure 3:**
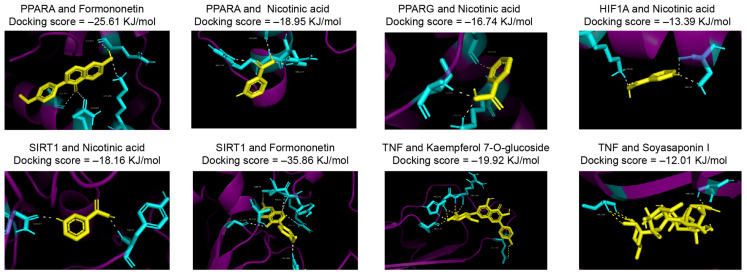
Molecular docking interactions between key targets with absorbed prototype metabolites in DBT.

In total, 717 targets associated with metabolic disorders, including ketosis, hyperketonemia, and lipid mobilization, were identified by referencing a public database (Figure 2A). After predicting the targets, 173 targets were identified within the absorbed prototype metabolites of DBT (Figure 2B). Forty shared proteins were determined at the intersection of targets between the disease and the botanical drug, with TNF, AKT1, PPARA, and PPARG identified as core interaction targets. KEGG pathway analysis indicated that the protein targets of the prototype metabolites in DBT mainly participated in the PPAR signaling pathway, glycerol–lipid metabolism, and cholesterol metabolism (Figure 2C). Centrality analysis of protein–protein interaction networks was conducted to further explore the significant targets of DBT intervention. By assessing the betweenness centrality and maximal clique centrality, five key target proteins associated with prototype metabolites were identified: PPARG, PPAGA, HIFIA, SIRT1, and TNF (Figure 2D). Additionally, these five proteins exhibited favorable docking patterns with the four metabolites in DBT (Figure 3). For example, PPARA formed hydrogen bonds with formononetin at positions ALA-441, GLN-445, LYS-448, and GLN-461, resulting in a docking score of −25.61 KJ/mol. The docking score between TNF and kaempferol 3-glucuronide was −19.92 KJ/mol, with eight hydrogen bonds formed at positions PRO-177, HIS-179, ARG-180, and GLU-187. The docking scores of nicotinic acid with PPARA, PPARG, HIFIA, and SIRT1 were −18.95 KJ/mol, −16.74 KJ/mol, −13.39 KJ/mol, and −18.16 KJ/mol, respectively.

### 3.4. DBT Intervention Alters the Composition of Fecal Microbiota

**Figure 4:**
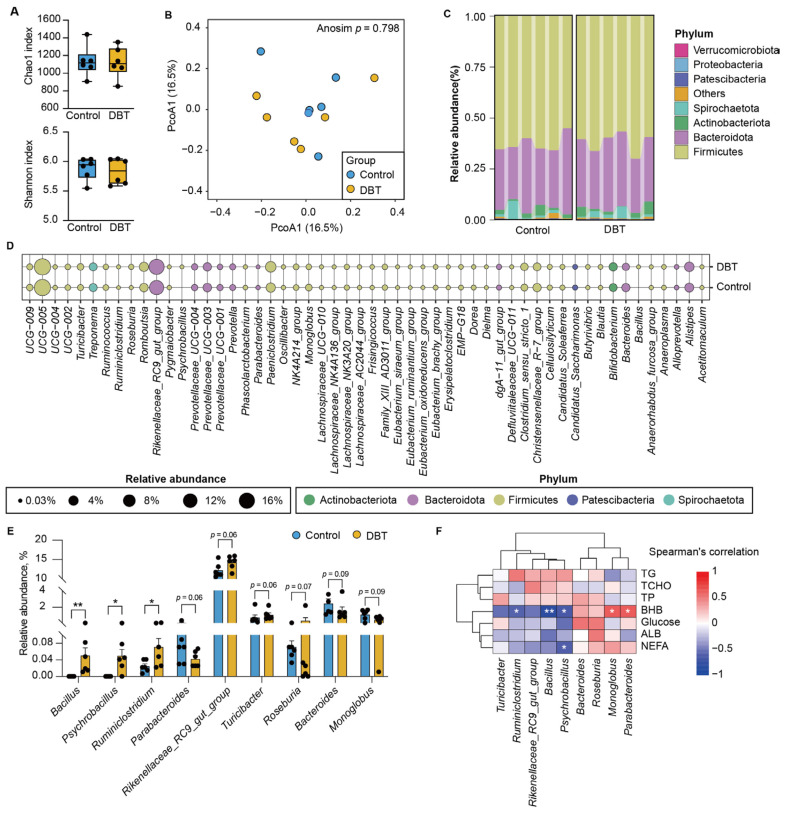
Danggui Buxue Tang altered the composition of the gut microbial community. (**A**) Alpha diversity, including Chao1 and Shannon index. (**B**) Principal coordinate analysis based on Bray-Curtis distance. (**C**) The relative abundance of phylum taxa. (**D**) The bubble plot displays the relative abundance (only > 0.1% is shown) of core genera taxa in feces between the DBT and control groups. (**E**) Differential analysis of the bacteria at the genus level based on Mann–Whitney U test. (**F**) The association between differential genera taxa and metabolic phenotypes was performed using Spearman’s coefficient. ** *p* < 0.01, 0.01 < * *p* < 0.05.

The alpha diversity of the fecal microbial community, as indicated by the Chao1 and Shannon indices, was not significantly different between the control and DBT groups (Figure 4A). Structural heterogeneity in fecal microbiota also showed no significant differences between the two groups (Figure 4B). The major taxa in the feces of dairy cows during the early lactation period were *UCG-005*, *Paeniclostridium*, *Clostridium sensu stricto 1*, and *Christensenellaceae_R-7*, all of which belong to Firmicutes (Figure 4C,D). Furthermore, *Rikenellaceae_RC9_gut_group*, *Alistipes*, *Prevotellaceae_UCG-003*, and *Bacteroides*, categorized under Bacteroidetes, exhibited higher relative abundances in the feces of dairy cows (Figure 4D). Compared with the control group, the relative abundance of *Rikenellaceae_RC9_gut_group* was increased (*p* = 0.06) and the relative abundance of *Bacteroides* (*p* = 0.09) was decreased in the DBT group (Figure 4E). The relative abundances of *Bacillus* (Cohen’s d = 1.54, 95% CI: 0.011–0.126), *Psychrobacillus* (Cohen’s d = 1.83, 95% CI: 0.00–0.108), and *Ruminiclostridium* (Cohen’s d = 1.24, 95% CI: 0.00–0.108) were significantly (*p* < 0.05) higher in the DBT group than in the control group with a larger effect size. In particular, increases in the relative abundances of *Bacillus*, *Psychrobacillus*, and *Ruminiclostridium* showed strong negative correlations with decreases in the level of circulating BHB (Figure 4F). *Psychrobacillus* abundance was also negatively correlated with serum NEFA levels. Moreover, the relative abundance of *Parabacteroides* and *Monoglobus* was lower in the DBT group than in the control group, with a strong positive correlation between these taxa and blood BHB levels.

**Figure 5:**
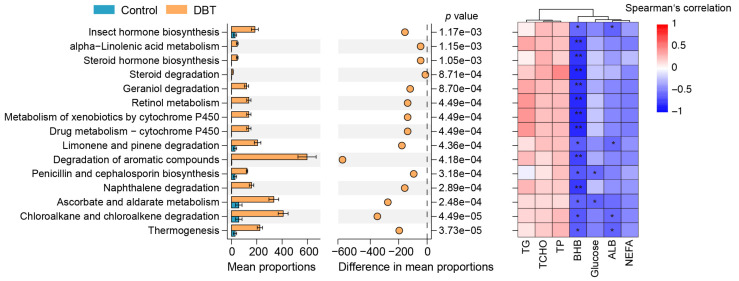
The differential analysis in predicated functions of gut microbiota and its correlation with metabolic phenotypes. ** *p* < 0.01, 0.01 < * *p* < 0.05.

To examine the effect of DBT on host metabolism by manipulating the gut microbiota, a predicted functional analysis of the gut microbiota was conducted. Alterations in 15 KEGG pathways were identified between the control and DBT groups (Figure 5). Compared to the control group, pathways such as alpha-linolenic acid metabolism, retinol metabolism, degradation of aromatic compounds, and ascorbate and aldarate metabolism were significantly (*p* < 0.05) upregulated in the DBT group, showing a strong negative correlation with the level of circulating BHB.

### 3.5. DBT Alters the Serum Metabolic Profiling of DAIRY Cows

**Figure 6:**
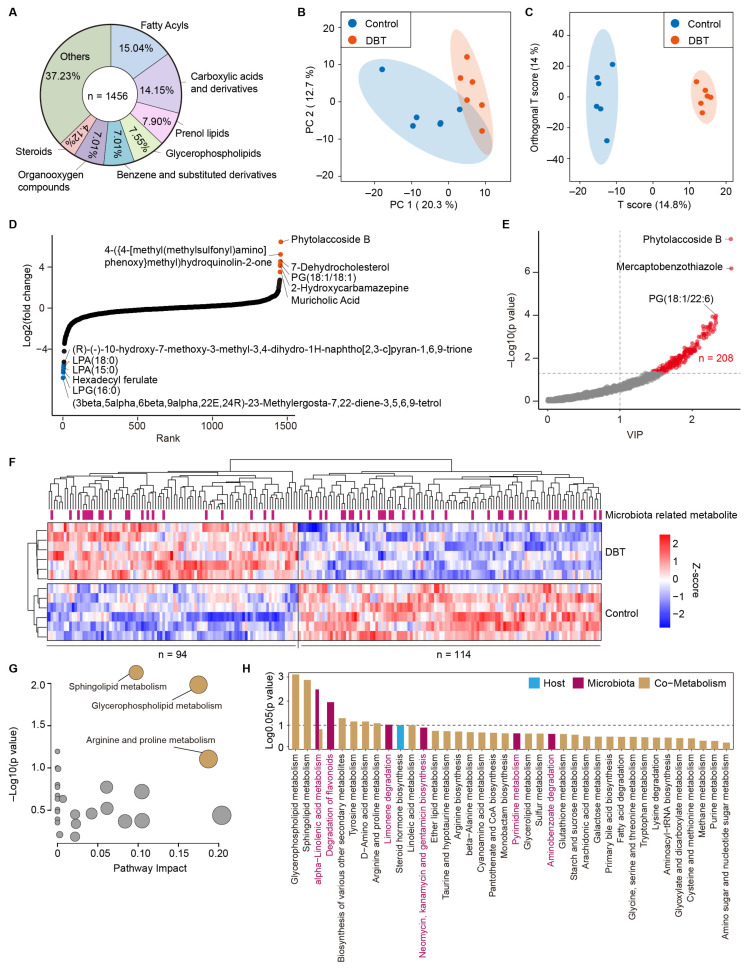
Danggui Buxue Tang alters the metabolic profiling in serum of postpartum dairy cows. (**A**) The classification of metabolites at the superclass level. (**B**) Principal component analysis. (**C**) Orthogonal partial least squares discriminant analysis (OPLS-DA). (**D**) The scatter plot shows the fold change (translated log_2_) of metabolites. Log_2_ (fold change) above 0 indicates a higher level of the metabolite in the DBT group than that in the control group; log_2_ (fold change) lower than 0 indicates a lower level of the metabolite in the DBT group. (**E**) Screening of differential metabolites based on *p*-value and VIP obtained from OPLS-DA. (**F**) The heatmap displays the differential metabolites in serum between the DBT and control groups. (**G**) The topology analysis of KEGG pathway. (**H**) The histogram shows the origin analysis of the KEGG level 3 pathway, including host, microbiota, and co-metabolism.

A total of 1456 metabolites were identified in the serum of dairy cows at DIM 7 using LC-MS/MS (Figure 6A). The major metabolites were fatty acids (15.04%), carboxylic acids (14.15%), propenol lipids (7.90%), and glycerophospholipids (7.55%). The principal component analysis and OPLS-DA score plots showed a distinct separation of the serum metabolome between the control and DBT groups (Figure 6B,C). The OPLS-DA model demonstrated excellent goodness of fit and predictive capability, as evidenced by the model validation parameters: R2Y = 0.99 and Q2 = 0.58. The fold-change analysis showed higher levels of phytolaccoside B, 7-dehydrocholesterol, phosphatidylglycerol (18:1/18:1), 2-hydroxycarbamazepine, and muricholic acid in the DBT group than those in the control group, whereas the levels of lysophosphatidic acid (18:0), lysophosphatidic acid (15:0), hexadecyl ferulate, and lysophosphatidylglycerol (16:0) were lower in the DBT group (Figure 6D). In total, 208 differential metabolites were identified between the control and DBT groups based on VIP > 1 and *p* < 0.05 criteria (Figure 6E), such as phytolaccoside B, mercaptobenzothiazole, and phosphatidylglycerol (18:1/22:6). Ninety-four serum metabolites were significantly increased in the DBT group compared with those in the control group (Figure 6F). The KEGG pathway analysis of differential metabolites indicated that glycerophospholipid metabolism, sphingolipid metabolism, and arginine and proline metabolism were significantly altered in dairy cows with DBT intervention (Figure 6G). Moreover, 57 metabolites related to the microbiota were identified using MetOrigin analysis, of which 5-methoxyindole-2-carboxylic acid and primin were microbial-specific metabolites. Three microbiota-related metabolic pathways were significantly altered in dairy cows treated with DBT, namely alpha-linolenic acid metabolism, flavonoid degradation, and limonene degradation (Figure 6H).

**Figure 7:**
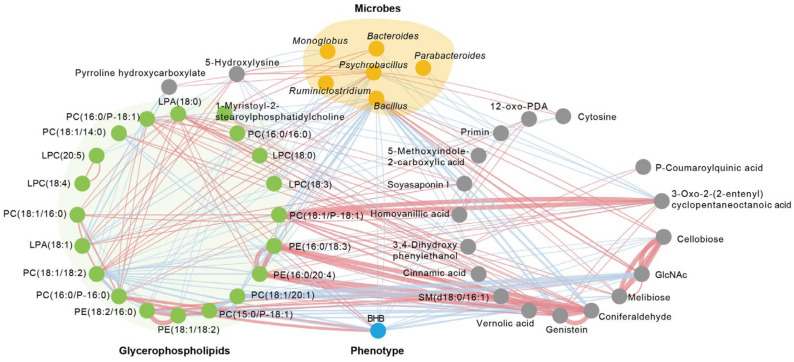
The network plot displays the relationships among key metabolic phenotypes, serum metabolites, and gut microbiota based on Spearman’s correlation analysis. The line color indicates positive correlations (red) and negative correlations (blue). The thickness of the line indicates the size of the correlation coefficient; only *p* values (FDR adjusted) < 0.05 are shown.

To investigate the relationship between the gut microbiota and host metabolism during DBT intervention, network analysis was performed to examine the correlations among gut microbes, differential metabolites, and clinical variables using Spearman’s coefficient (Figure 7). Glycerophospholipids emerged as key metabolites linking microbes to BHB, notably phosphatidylcholine (PC, 18:1/P-18:1), phosphatidylethanolamine (PE, 16:0/18:3), PE (16:0/20:4), and PC (18:1/20:1), which exhibit a higher number of edges.

## 4. Discussion

Dairy cows typically develop various diseases during the postpartum period, and metabolic disorders are significant factors in the incidence of these diseases [3]. This study showed that DBT intervention significantly reduced the levels of NEFA and BHB in the blood of postpartum cows; this may be related to the absorbed prototype metabolites of DBT, including formononetin, kaempferol 3-glucuronide, nicotinic acid, and soyasaponin I. Based on the network pharmacology analysis, PPAR, SIRT1, and TNF may be potential pathways through which DBT alleviates metabolic stress during the postpartum period. To further explore the regulatory pathways, this study showed that DBT altered the composition of the gut microbiota, particularly by increasing the relative abundance of *Bacillus*, *Psychrobacillus*, and *Ruminiclostridium*, changes which are related to a decrease in BHB levels. Glycerophospholipid metabolism serves as an important metabolic pathway linking the gut microbiota to clinical phenotypes that were determined using integrated analysis of blood metabolomics. These findings provide new insights into the alleviation of metabolic stress in transition dairy cows.

Excessive lipid mobilization has long been regarded as a potential contributing factor to the occurrence of periparturient diseases in dairy cows [23,24]. Typically, the level of circulating NEFA peaks after calving and then gradually declines during early lactation; but, in cows experiencing metabolic maladaptation, NEFA levels may continue to rise [25]. Additionally, NEFA are transported to the liver, where they are further metabolized to BHB via the beta-oxidation pathway. Although low concentrations of BHB can be utilized by extrahepatic tissues as an alternative fuel, elevated concentrations can provoke inflammatory responses and insulin resistance within the organism [26,27]. In this study, DBT can reduce the circulating NEFA and BHB levels in dairy cows during the postpartum period. As the primary component of DBT, *Astragalus* supplementation significantly reduces circulating levels of NEFA and TG through the regulation of key hepatic genes involved in lipid metabolism, including *PPARα*, *CPT1*, and *SREBP-1* [28]. A previous study also suggested that the *Astragalus* polysaccharide plays a beneficial role in the metabolic health of dairy cows by regulating glucose and amino acid metabolism [29]. Furthermore, Tian et al. [30] indicated that the *Angelica* polysaccharide decreases the levels of inflammatory mediators in dairy cows with laminitis by inhibiting the NF-κB signaling pathway. As major active compounds of natural products, polysaccharides enhance glucose and lipid homeostasis by alleviating oxidative stress [31]. In addition to the polysaccharides, a number of compounds of DBT have been identified, including astragaloside, formononetin, ferulic acid, vanillic acid, and isoflavanone [17]. Astragaloside IV and senkyunolide in DBT are key ingredients regulating immunity and hematopoiesis, as indicated by network pharmacology and molecular docking analyses [32]. Unlike conventional database searches, the present study determined five absorbed prototype metabolites of DBT using serum pharmacochemistry: formononetin, kaempferol 3-glucuronide, nicotinic acid, amygdalin, and soyasaponin I. Formononetin, derived from *Astragali Radix*, has been shown to alleviate the metabolic disorder of lipids by modulating the SIRT1 and PPARα pathways [33,34]. Among the isoflavone group, formononetin exhibits moderate bioavailability in ruminants, primarily undergoing extensive phase II metabolism to form glucuronide and sulfate conjugates [35]. Its metabolic stability is relatively high in dairy cows, allowing for sustained biological activity [36]. Nicotinic acid, a bioactive component present in both *Astragali radix* and *Angelica sinensis radix*, shows exceptional bioavailability in ruminants, characterized by rapid absorption and systemic distribution [37]. Numerous studies have recommended dietary supplementation of nicotinic acid as an effective strategy to mitigate heat stress and regulate lipid mobilization in dairy cows [38,39]. Regarding soyasaponins, these triterpenoid compounds display limited systemic bioavailability due to their substantial molecular structure and extensive microbial biotransformation in the ruminal environment [40]. Their biological effects are predominantly mediated through microbial-derived metabolites and localized actions within the gastrointestinal tract. Similarly, kaempferol 3-glucuronide, a flavonoid conjugate, demonstrates restricted membrane permeability but can be enzymatically hydrolyzed to its aglycone form by gut microbial enzymes [41]. From a functional perspective, kaempferol 3-glucuronate can decrease pro-inflammatory mediators such as interleukin-1β and leukotriene B4 by inhibiting the NF-κB signaling pathway [42]. The inflammatory response and oxidative stress during the transition period play a contributory role in lipid mobilization in dairy cows [43]. Comparative cross-sectional studies have demonstrated significant elevation of pro-inflammatory factors, including TNF-α, interleukin-6, and serum amyloid A, in cows with elevated circulating levels of NEFA and BHB [44,45,46]. Our findings identify TNF as a key regulatory target of DBT in dairy cattle, suggesting that this intervention may mitigate postpartum lipid mobilization by attenuating inflammatory responses.

Gut microbiota plays an important role in regulating metabolic homeostasis and drug metabolism [13,47]. Gut microbiota influences the efficacy of DBT and the transport of ononin to formononetin via gut microbiota-mediated deglycosylation [48]. Additionally, studies have demonstrated that the relative abundance of *Bacteroides* correlates with the absorption of metabolites from DBT. Du et al. [10] study show that DBT increased the relative abundance of *Bacteroides* in the feces of mice following antibiotic treatment, whereas the relative abundance of *Ruminiclostridium* was not significantly different. The results of the gut microbial community analysis showed that DBT significantly increased the relative abundance of *Ruminiclostridium* and *Bacillus* in postpartum dairy cows. *Bacillus*, a genus of Gram-positive bacteria, is typically considered beneficial because of its association with immune homeostasis [49]. A nutritional intervention study showed that diets supplemented with *Bacillus* could enhance milk yield and improve milk composition in dairy cows [50]. Terré et al. [51] reported that the feed efficiency of lactating dairy cows increased significantly when they were fed a *Bacillus*-mixed diet. Regarding metabolic health regulation, emerging evidence demonstrates that *Bacillus* plays a crucial role in ameliorating chronic low-grade inflammation and metabolic dysfunction by suppressing pro-inflammatory cytokines, including TNF-α and interleukin-6 [52]. Furthermore, a previous study has established that *Bacillus* supplementation effectively reduces serum triglyceride levels and alleviates metabolic syndrome-related parameters [53]. Unlike conventional probiotics, *Bacillus spores* exhibit exceptional resistance to environmental stressors, including heat, gastric acid, and oxygen exposure. This stability allows effective colonization and sustained metabolic benefits, such as long-term improvements in lipid profiles and glycemic control [54,55]. Additionally, the relative abundance of *Parabacteroides* and *Monoglobus* in the feces was increased in dairy cows receiving DBT. A recent study has reported that *Parabacteroides* in the intestine are closely associated with the development of metabolic diseases, including obesity, fatty liver disease, and diabetes mellitus [56]. Gut commensal *Parabacteroides distasonis*, belonging to *Parabacteroides*, is regarded as a beneficial bacterium that positively regulates the inflammatory response and insulin resistance [57,58]. However, a potential pathogenic role of *Parabacteroides distasonis* has been identified [59]. Previous studies have indicated that the relative abundance of *Monoglobus* is possibly associated with the potential for certain diseases [60]. DBT exerted a positive regulatory effect on host health by influencing the composition of the gut microbial community. However, one limitation of this study is that the small sample size may not sufficiently account for the influence of potential confounding factors on gut microbiota.

In the present study, changes in glycerophospholipid metabolism following DBT intervention were strongly correlated with a decrease in circulating BHB levels during the postpartum period. The dysregulation of glycerophospholipid metabolism and its metabolites is considered to contribute to disease development [61,62]. Previous research has shown significant alterations in glycerophospholipid metabolism in healthy dairy cows transitioning from late gestation to early lactation [63,64]. Zheng et al. [65] conducted a study on the urinary metabolite signatures of dairy cows with ketosis and the levels of PC, including PC (32:2), PC (38:3), PC (30:2), and PC (36:0), decreased in cows diagnosed with ketosis. Huang et al. [66] also reported that the levels of several glycerophospholipids decreased in dairy cows experiencing ketosis, as identified by plasma metabolomic analysis. Common glycerophospholipids such as PE and PC are derived from diacylglycerols via enzymatic reactions. In present study, serum levels of PE (16:0/20:4), PE (16:0/18:3), PC (16:0/16:0), and PC (18:0/18:2) decreased in dairy cows treated with DBT, whereas the levels of PE (18:2/16:0), PE (18:1/18:2), PC (18:1/18:2), and PC (18:1/14:0) increased. Consistent with the present findings, a previous study has indicated that the plasma levels of PC (16:0/18:2), PC (16:0/18:1), PC (16:0/20:4), and PE (16:0/22:6) are markedly elevated in dairy goats with hyperketonemia [67]. Zhao et al. [68] study indicated increased levels of PC (17:0/18:2), PE (18:1/18:2), PC (16:0/20:4), and PE (18:0/18:1) in the raw milk of dairy cows with sub-ketosis, suggesting that the health status of dairy cows influences glycerophospholipid metabolism. Furthermore, the levels of lysophosphatidylcholine (LPC), including LPC (20:5), LPC (18:4), LPC (18:3), and LPC (18:0), were decreased in the DBT group. PC is metabolized to LPC through the action of phospholipases; yet, an imbalance between PC and LPC has been associated with inflammatory responses [69]. Although numerous studies have confirmed the presence of altered glycerophospholipid levels in metabolic disorders, the precise mechanisms by which glycerophospholipids affect metabolic health require further investigation. Regarding alternative nutritional and phytochemical interventions, rumen-protected choline, propylene glycol, and resveratrol have been widely investigated for their potential to regulate metabolic disorders in dairy cows. Potts et al. [70] demonstrated that rumen-protected choline supplementation significantly modulates glycerophospholipid metabolism during the periparturient period by regulating the expression of hepatic lipid metabolism genes. Unlike choline, propylene glycol is primarily used for the management of metabolic diseases, particularly ketosis and left displaced abomasum [71]. Clinical trials have shown that supplementation with 150g/head/day of propylene glycol effectively reduces circulating NEFA and BHB levels in postpartum cows [72]. However, prolonged administration of propylene glycol appears to offer limited benefits [73]. Resveratrol, a polyphenolic compound, has shown promise as a feed additive through multiple mechanisms, including inhibition of lipogenesis, reduction of pro-inflammatory cytokines, and enhancement of antioxidant capacity. Despite these benefits, its application in ruminants is limited by poor bioavailability due to extensive microbial degradation in the rumen [74]. Comparative analysis suggests that DBT may offer several advantages over these supplements in terms of cost-effectiveness, safety, and bioavailability.

## 5. Conclusions

Five absorbed prototype metabolites of DBT were identified, all of which correlated with decreased levels of circulating NEFA and BHB in postpartum dairy cows. Furthermore, DBT altered the gut microbial composition and glycerophospholipid metabolism, indicating its potential role in regulating the health of dairy cows during early lactation by alleviating metabolic stress. These DBTs are a cost-effective, natural alternative to conventional metabolic modifiers in dairy production. Based on our findings, we recommend implementing DBT supplementation during the postpartum period, accompanied by regular monitoring of liver function and milk composition, to optimize dosage regimens. However, comprehensive longitudinal studies should be pursued to further validate and extend these findings: (1) long-term effects of DBT supplementation on both production performance and reproductive efficiency in dairy cows, (2) large-scale in vivo validation studies, and (3) targeted metabolomics approaches to elucidate the key regulatory pathways of individual bioactive components within DBT.

## Figures and Tables

**Table 1:** The serum variable levels of the control and DBT cows at days 0 and 7.

Items	Day 0		Day 7		SEM	Percentage Change at Day 7	P _Time_	P _Group_	P*_Time × group_*
	Control	DBT	Control	DBT					
NEFA (mmol/L)	0.63	0.61	0.51	0.32 #	0.05	−37.25%	0.005	0.11	0.24
BHB (mmol/L)	0.37	0.33	1.07	0.73 *	0.08	−31.78%	<0.001	<0.001	0.004
TG (mmol/L)	0.14	0.15	0.13	0.17	0.01	+30.77%	0.08	0.73	0.26
TCHO (mmol/L)	1.88	1.83	2.04	2.29	0.11	+12.25%	0.1	0.58	0.42
Glucose (mmol/L)	4.44	4.78	3.58	3.3	0.2	−7.82%	<0.0001	0.91	0.19
ALB (g/L)	29.42	27.41	29.31	31.36	1.78	+6.99%	0.54	0.99	0.52
TP (g/L)	61.78	62.31	63.8	64.46	1.35	+1.03%	0.8	0.39	0.98

Note: Percentage change was calculated as: [(DBT group mean − Control group mean)/Control group mean] × 100. NEFA, non-esterified fatty acids. BHB, beta-hydroxybutyrate. TG, triglycerides. TCHO, total cholesterol. ALB, albumin. TP, total protein. * *p* < 0.05, 0.05 < # *p* < 0.1.

**Table 2:** The identification information of prototype metabolites in DBT.

Name	Formula	RT (min)	Calculated (*m*/*z*)	Adduct	Error (ppm)	MS/MS Spectrum	Origin
Amygdalin	C20H27NO11	11.915	502.15604	[M + FA-H]-	1.10	59.0120; 71.01202; 89.02302; 101.02272; 113.02249; 179.05492; 221.06511	DG
Formononetin	C16H12O4	17.788	267.06561	[M-H]-	2.62	132.0206; 223.03844; 252.04068	HQ
Kaempferol 3-glucuronide	C21H18O12	13.185	461.07217	[M-H]-	0.74	59.01207; 85.02802; 113.02233; 175.02414; 285.03918; 327.05161	HQ
Nicotinic acid	C6H5NO2	1.437	124.03951	[M + H]+	1.85	56.05018; 68.05043; 78.03463; 82.06587; 96.04481; 109.05316	DG, HQ
Soyasaponin I	C48H78O18	17.571	987.51794	[M + FA-H]-	0.92	85.02788; 143.03345; 205.0695;457.36401; 615.39209; 733.4502; 879.51715; 923.50751; 941.51624	HQ

## Data Availability

The original contributions presented in this study are included in the article. Further inquiries can be directed to the corresponding authors.
